# Anti-*Toxoplasma* IgG assays: What performances for what purpose? A systematic review

**DOI:** 10.1051/parasite/2021035

**Published:** 2021-04-26

**Authors:** Florence Robert-Gangneux, Hélène Guegan

**Affiliations:** 1 Université de Rennes, CHU Rennes, Inserm, EHESP, Irset (Institut de Recherche en Santé Environnement Travail), UMR_S 1085 35000 Rennes France

**Keywords:** Anti-*Toxoplasma* IgG, Diagnosis, Serology, Toxoplasmosis, Sensitivity, Specificity

## Abstract

Chronic infection with *Toxoplasma gondii* is attested by the detection of specific anti-*Toxoplasma* IgG. A wide panel of serologic methods is currently marketed, and the most suitable method should be chosen according to the laboratory resources and the screened population. This systematic review of evaluation studies aimed at establishing an overview of the performances, i.e. sensitivity, specificity, positive predictive value (PPV) and negative predictive value (NPV) of marketed anti-*Toxoplasma* IgG assays, and discussing their technical characteristics to guide further choice for routine diagnostic use. According to PRISMA guidelines, the search performed in PubMed and Web of Science databases recovered 826 studies, of which 17 were ultimately included. Twenty commercial anti-*Toxoplasma* IgG assays were evaluated, in comparison with an accepted reference method. Most of them were enzyme-immunoassays (EIAs, *n* = 12), followed by agglutination tests (*n* = 4), immunochromatographic tests (*n* = 3), and a Western-Blot assay (WB, *n* = 1). The mean sensitivity of IgG assays ranged from 89.7% to 100% for standard titers and from 13.4% to 99.2% for low IgG titers. A few studies pointed out the ability of some methods, especially WB to detect IgG early after primary infection. The specificity of IgG assays was generally high, ranging from 91.3% to 100%; and higher than 99% for most EIA assays. The PPV was not a discriminant indicator among methods, whereas significant disparities (87.5%–100%) were reported among NPVs, a key-parameter assessing the ability to definitively rule out a *Toxoplasma* infection in patients at-risk for opportunistic infections.

## Abstract (fr)

L’infection chronique à *Toxoplasma gondii* est attestée par la détection d’IgG anti-*Toxoplasma* spécifiques. Un large panel de méthodes sérologiques est actuellement commercialisé, et le choix d’une méthode doit être adapté aux ressources du laboratoire ainsi qu’à la population ciblée. Cette revue systématique des études d’évaluation visait à établir une vue d’sensemble des performances, c’est-à-dire la sensibilité, la spécificité, la valeur prédictive positive (VPP) et la valeur prédictive négative (VPN) des kits commercialisés pour la détection d’IgG anti-*Toxoplasma*, et à discuter leurs caractéristiques techniques pour guider le choix pour un usage diagnostique de routine. Selon les directives PRISMA, la recherche effectuée dans les bases de données PubMed et Web of Science a permis de retrouver 826 études, dont 17 ont été définitivement incluses. Vingt dosages commerciaux d’IgG anti-*Toxoplasma* ont été évalués, en comparaison avec une méthode de référence. La plupart des tests étaient des méthodes de dosage immuno-enzymatique (*n* = 12), d’agglutination (*n* = 4), immunochromatographiques (*n* = 3) et de Western-Blot (*n* = 1). La sensibilité moyenne des dosages IgG variait de 89,7 à 100 % pour les titres standards et de 13,4 % à 99,2 % pour les faibles titres d’IgG. Quelques études ont souligné la capacité de certaines méthodes, en particulier le Western-Blot, à détecter les IgG au cours d’une primo-infection. La spécificité des tests IgG était généralement élevée, allant de 91,3 % à 100, et supérieure à 99 % pour la plupart des tests immuno-enzymatiques. La VPP n’était pas un indicateur discriminant entre les méthodes, alors que des disparités significatives (87,5 % à 100 %) ont été rapportées entre les VPN, un paramètre-clé reflétant la capacité d’un test à éliminer formellement une toxoplasmose chez les patients à risque d’infections opportunistes.

## Introduction

Toxoplasmosis is a foodborne or waterborne protozoan infection, with an estimated seroprevalence of 30% worldwide [20]. However, there are huge differences in prevalence rates among geographical areas, mainly in relation to climate, dietary and social habits, and socioeconomic levels. Infection with *Toxoplasma gondii* is often unnoticeable and after a first step of systemic dissemination, the parasites become encysted and remain lifelong in various anatomic sites, notably the brain, the muscles, and the retina. Serology is an important tool for the diagnosis of toxoplasmosis and is widely used to determine whether a pregnant woman is at risk of primary infection during pregnancy or if an immunocompromized patient is at risk of *Toxoplasma* reactivation. Importantly, toxoplasmosis can lead to congenital infection when acquired by a non-immune pregnant woman, with a rate of transmission and severity depending on the trimester of pregnancy at maternal infection. Additionally, encysted parasites can reactivate in case of immune suppression (HIV infection, transplantation, immunosuppressive therapies, etc.) and cause encephalitis, retinochoroiditis, or disseminated infection with a high mortality rate [6, 21]. Knowledge of the immune status allows (i) if negative, to provide targeted clinical counseling to avoid infection, and (ii) if positive, to include toxoplasmosis among possible opportunistic infections in immunocompromized patients with evocative clinical signs, or to prescribe chemoprophylaxis [6]. Usually, *Toxoplasma* serology relies on the detection of both specific IgG and IgM, allowing precise interpretation of results, taking advantage of the kinetics of isotypes detection. However, anti-*Toxoplasma* IgG is the key parameter to indicate past infection or to confirm primary infection, as the sole detection of anti-*Toxoplasma* IgM is not conclusive. Therefore, the specificity and sensitivity of anti-*Toxoplasma* IgG assays is crucial. Many IgG assays are marketed worldwide, either manual or automated, and are based on various detection methods including agglutination assays, western-blot assays, enzyme immunoassays (EIAs), and immunofluorescence assays. They have variable performances and thresholds of detection, although most of them are supposed to be standardized upon an international standard [17, 27]. More recently, rapid diagnostic tests (RDTs) have also been developed.

The choice of the technique may depend on the situation and the targeted goal: Will the assay be used in accredited laboratories of high-income countries? Will it be adapted to climate and primary care structures of low-income countries? This systematic review aimed at compiling all evaluation studies on anti-*Toxoplasma* IgG assays, to provide an accurate view on the performance and practicability of marketed tests and help guide the choice of an IgG assay.

## Methods

The study was performed according to the Preferred Reporting Items for Systematic review and Meta-Analyses (PRISMA) guidelines. Published literature was searched for in the PubMed database, combining medical subject headings (MeSH) terms as follows: (((“Toxoplasmosis/diagnosis”[Mesh]) AND “Serologic Tests”[Mesh])) NOT “Toxoplasmosis, Congenital”[Mesh], and restricted to “humans”, “abstract available”, “English” language and timespan 1990–2020 (October 28th). Other searches were done using the following keywords: anti-Toxoplasma IgG, toxoplasmosis serology or toxoplasmosis assay in PubMed (restricted to the fields “title/abstract”) and Web of Science databases, applying the same restrictions. For the Web of Science database, the search terms were “toxoplasmosis serology” or “anti-toxoplasma IgG” or “toxoplasmosis assay”, and results were further focused on the following research areas: “Parasitology”, “Infectious Diseases”, “Microbiology” and “Medical Laboratory Technology” and refined with “evaluation”, “comparison”, or “performance” terms. All references retrieved were screened one by one, and only studies evaluating the performance of commercial serological assays by comparison to a well-recognized reference method were included for analysis.

### Data extraction

All selected articles were studied for further inclusion. Data collected were: first author name, publication year, country of study, study design, population studied, sample size, diagnostic method evaluated, reference method used, results of sensitivity, specificity, and positive predictive value (PPV) and negative predictive value (NPV), if relevant.

### Statistics

Sensitivity was calculated from the results obtained for all positive sera, referring to the reference method used. Results obtained in the Grey Zone (GZ) were considered negative results, unless they were excluded by the authors and detailed results were not provided (one study). Sensitivity was calculated as: number of true positive results/number of seropositive patients. Specificity was calculated as follows: number of negative results/number of seronegative patients + number of false-positive results. PPV was calculated as the number of true-positive results/number of true-positive results + number of false-positive results. NPV was calculated as the number of true-negative results/number of true-negative results + number of false-negative results. When needed, sensitivity, specificity, PPV or NPV were re-calculated from the authors’ data to allow homogeneous comparison among studies regarding GZ results.

When several studies evaluated the same assay, results for sensitivity and specificity were presented as mean and range. Wherever appropriate, sensitivity obtained on low-IgG sample collections was presented separately.

Graphs were constructed using GraphPad Prism v6 software.

## Results

### Articles included

**Figure 1:**
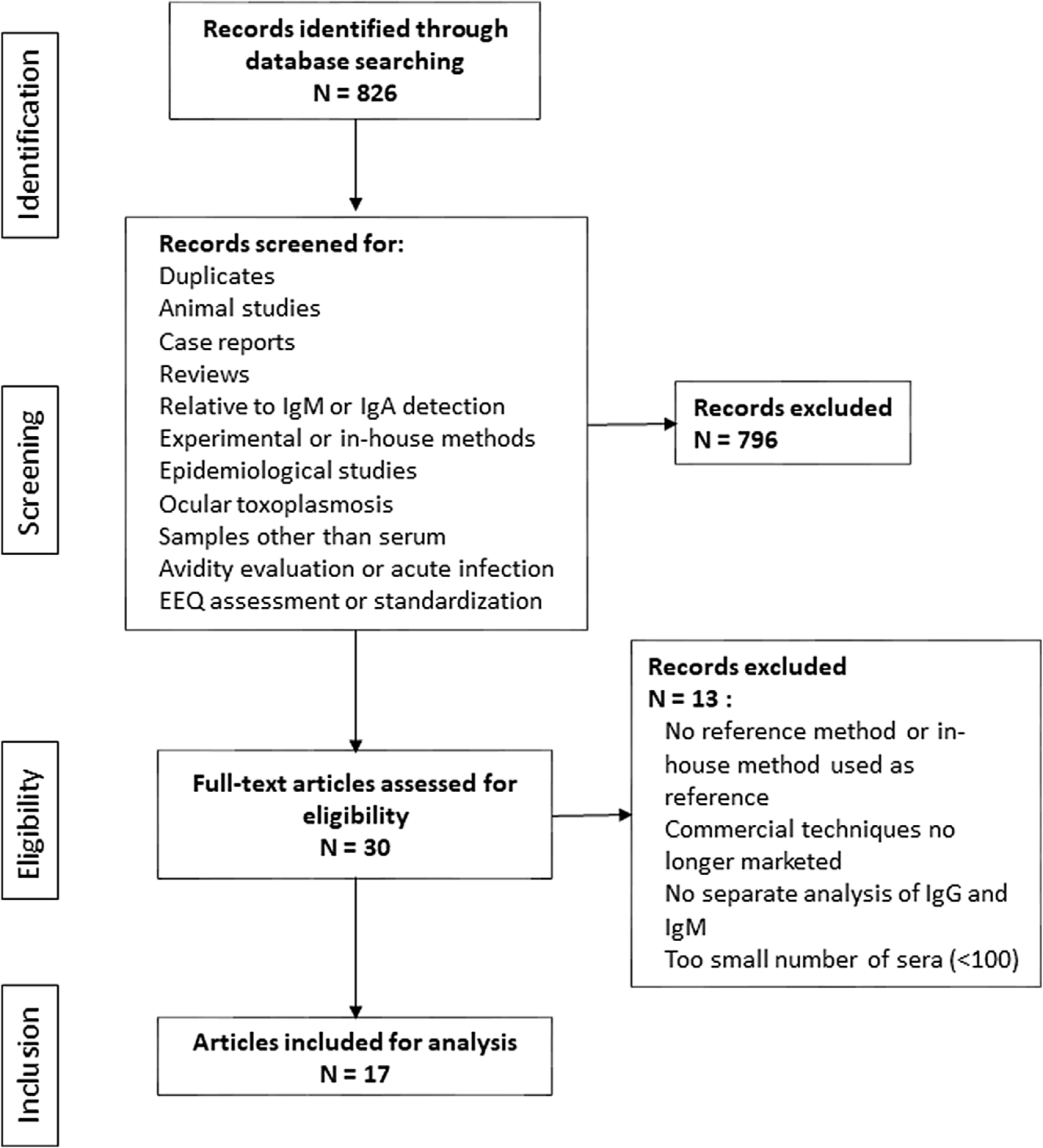
Flow chart of article selection for review.

The results of the various searches are described in Supplementary Table 1. All studies dealing with prevalence analysis, animal studies, experimental or research methods and simple correlation studies were discarded. After careful reading of 826 titles/abstracts, 30 eligible articles were included for thorough reading and analysis. Thirteen articles were further excluded, either because they provided vague results or merged IgG/IgM results (three studies), or used an in-house or another EIA method as the gold standard or no reference method at all (six studies), or used methods which are no longer marketed (three studies), or included a too small number of sera (<100) (one study). Finally, 17 studies were included in the analysis (Fig. 1).

### Methods evaluated

Overall, the 17 studies evaluated 28 methods for the detection of anti-*Toxoplasma* IgG, of which 8 were no longer marketed in 2020; therefore, we focused on results obtained on the 20 remaining assays listed in Table 1. They consisted of agglutination assays (latex agglutination or hemagglutination, *n* = 4), EIA (*n* = 12) using various detection systems or matrices (microplate enzyme-linked immuno-sorbent assay (ELISA), microparticle enzyme immunoassay (MEIA), enzyme-linked fluorescent assays (ELFA), chemiluminescent microparticle immunoassay (CMIA), electrochemiluminescence immunoassay (ECLIA)), western-blot assay (*n* = 1) and RDT based on immunochromatographic assays (ICT, *n* = 3). There was no evaluation of immunofluorescence assays. Half of the assays were evaluated in only one study; the most frequently evaluated assays were Architect Toxo IgG^®^, Elecsys Toxo IgG^®^ and Vidas Toxo IgGII^®^ (Table 1). Most studies were based on a selection of sera from a bank, while only three studies included consecutive non-selected sera [12–14], of which one mixed both selected and prospectively included sera [13]. The dye-test (DT) was the most frequently used method to confirm discrepant results between compared assays (10 studies), associated with immunofluorescence assay (IFA) in three studies. The WB Toxo IgGII^®^ assay was used as a reference method in six studies. For the two remaining studies [19, 23], the reference method used was a combination of several immunoassays.

**Table 1:** *Toxoplasma* IgG commercial methods included: type of assay, number of evaluation studies, and population studied.

Technique	Firm	Type of method	Threshold (IU/mL)	No of studies	Population studied	No of sera	No of IgG-positive	References
Access Toxo IgG II^®^	Beckman Coulter Inc.	MEIA, automated	7.5 ≤ *x* < 10.5	2	Pregnant women	760	257	[4, 14]
					Low IgG			
Advia Centaur Toxo IgG^®^	Siemens Healthineers	CMIA, automated£	6.4 < *x* ≤ 10	1	Miscellaneous	406	207	[24]
Architect Toxo IgG^®^	Abbott	MEIA, automated£	1.6 ≤ *x* < 3	5	Pregnant women	2992	773	[4, 7, 13, 16, 24]
					Low IgG			
					Miscellaneous			
AxSYM Toxo IgG^®^	Abbott	MEIA, automated£	2 ≤ *x* < 3	3	Pregnant women	1555	638	[7, 14, 24]
					Miscellaneous			
BioPlex 2200 ToRCH IgG/IgM^®^	BioRad	MFA, automated	9 ≤ *x* < 12	1	Miscellaneous	162	139	[9]
Elecsys Toxo IgG^®^	Roche Diagnostics	ECLIA, automated£	1 ≤ *x* < 30	5	Pregnant women	2214	1398	[4, 14, 18, 23, 24]
					Low IgG			
					Miscellaneous			
Immulite 2000 anti-Toxoplasma IgG^®^	Siemens Healthineers	MEIA, automated	6 ≤ *x* < 8	2	Miscellaneous	655	348	[14, 19]
					Pregnant women			
Liaison Toxo IgG II^®^	Diasorin	CMIA, automated£	7.2 ≤ *x* < 8.8	4	Low IgG	1381	460	[4, 16, 23, 24]
					Miscellaneous			
OnSite Toxo IgG/IgM Combo Rapid Test^®^	CTK Biotech	ICT, manual£		1	Miscellaneous	310	170	[8]
Pastorex^®^	BioRad	Agglutination£		1	Miscellaneous	589	344	[26]
Platelia Toxo IgG^®^	BioRad	Microplate ELISA, automated£	6 ≤ *x* < 8	3	Low IgG	1436	491	[4, 10, 24]
					Miscellaneous			
TGS TA Toxo IgG/IDS-iSYS^®^	TGS Technogenetics	MEIA, automated	>1.5	2	Low IgG	1137	319	[4, 12]
					Miscellaneous			
Toxo IgG/IgM Rapid Test^®^	Biopanda Reagents	ICT, manual£		1	Miscellaneous	310	170	[8]
Toxocell^®^	Biokit	Agglutination£		1	Miscellaneous	589	344	[26]
Toxo HAI^®^	Fumouze	Hemagglutination£		1	Miscellaneous	589	344	[26]
Toxolatex^®^	Fumouze	Agglutination£		1	Miscellaneous	589	344	[26]
Toxoplasma ICT IG/IgM^®^	LDBio	ICT, manual£		3	Low IgG	1492	559	[2, 8, 13]
					Miscellaneous			
Vidas Toxo IgG^®^	BioMérieux	ELFA, automated£	4 ≤ *x* < 8	7	Low IgG	3368	1093	[4, 7, 10, 14, 16, 23, 24]
					Miscellaneous			
					Pregnant women			
Vitros ECiQ Toxoplasma IgG^®^	Ortho Diagnostics	CLIA£	4 ≤ *x* < 8	1	Pregnant women	719	551	[11]
WB Toxo IgGII^®^	LDBio	WB, manual£		1	Low IgG	569	162£	[5]
					Miscellaneous			

£ With comparison to Dye-Test available.

ELISA: enzyme-linked immuno-sorbent assay, MEIA: microparticle enzyme immunoassay, ELFA: enzyme-linked fluorescent assay, CLIA: chemiluminescence immunoassay, CMIA: chemiluminescent microparticle immunoassay, ECLIA: electrochemiluminescence immunoassay, MFA: Multiplex fluorescence assay, WB: western-blot assay, ICT: immunochromatographic assay.

### Sensitivity

**Figure 2:**
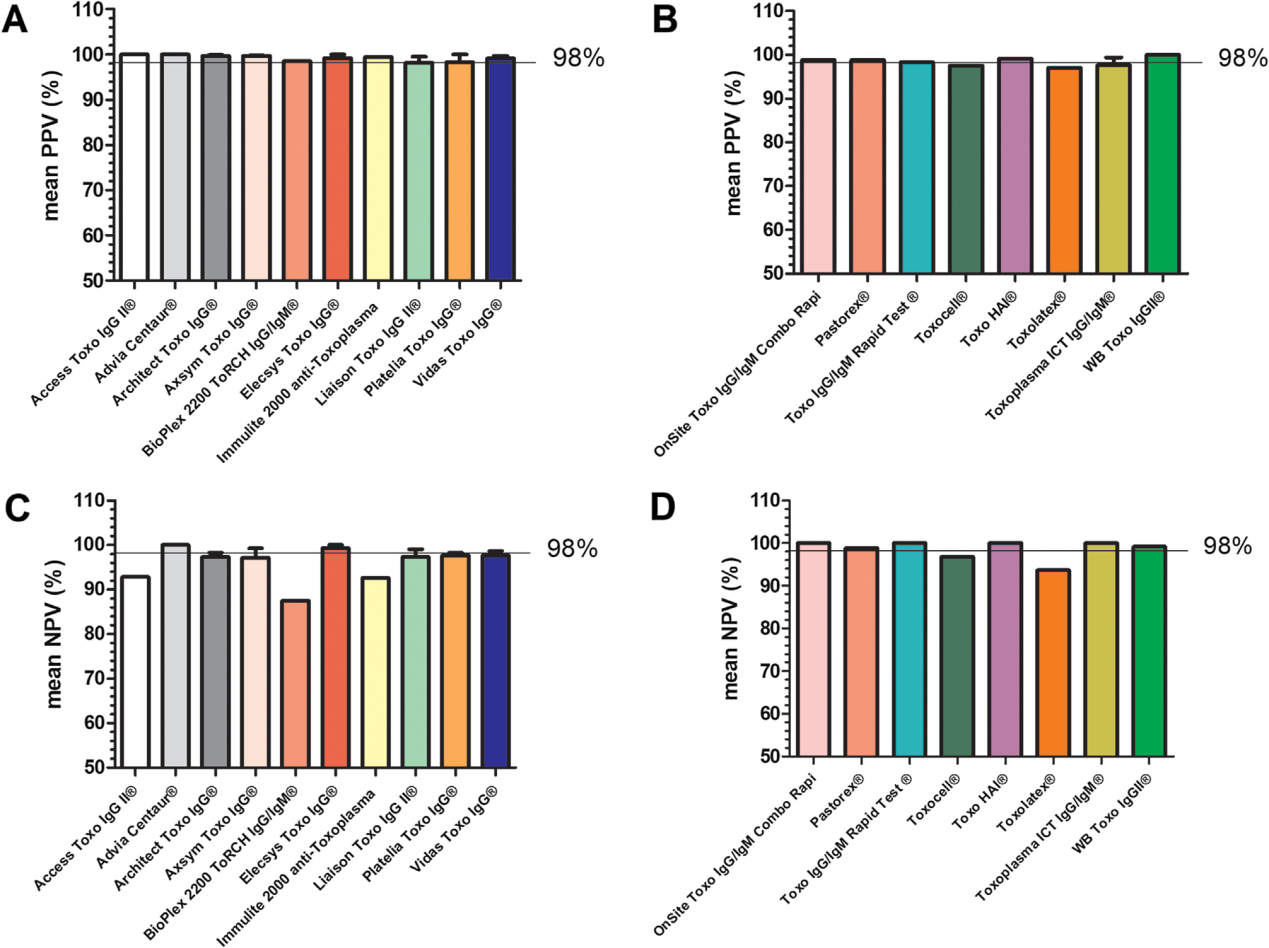
Positive predictive value (PPV) and negative predictive value (NPV) calculated for 10 anti-*Toxoplasma* IgG EIA assays (A, C) and 8 manual assays (B, D) included in the study.

The mean sensitivity of IgG assays on routine sera ranged from 89.7% to 100% (Table 2). Eleven out of 20 assays had sensitivity ≥97%. The mean sensitivity of each test is represented in Figure 2. Some manual methods had similar sensitivity rates to EIA. Studies evaluating the ability to detect low IgG titers were less numerous and yielded sensitivities ranging from 13.4% to 99.2% [4, 5, 13, 23, 24, 26]. The EIA assays displaying the poorest and the highest sensitivity in this setting were the Access Toxo IgGII^®^ and the Elecsys Toxo IgG^®^, respectively (Table 2). Among manual methods, the WB Toxo IgG II^®^ had the best sensitivity (99.2%) to detect low IgG titers. Among agglutination tests, the Toxo-HAI^®^ displayed the best sensitivity both on standard and low IgG titers (100% and 97%, respectively), but yielded 4.3% false-positive results with sera of patients with various interfering diseases [26]. Other agglutination assays (Toxolatex^®^, Toxocell^®^, and Pastorex Toxo^®^) performed well on standard IgG titers but not on low IgG detection (51.5%–66.7%) (Table 2). RDTs (OnSite Toxo IgG/IgM Combo Rapid Test^®^, Toxo IgG/IgM Rapid Test^®^ Biopanda, and Toxoplasma ICT IgG/IgM^®^ LDBio) showed very good sensitivity, but two of them were evaluated only in the standard situation. Their performance to detect low IgG titers therefore needs further evaluation.

**Table 2:** Sensitivity, Specificity, PPV and NPV of anti-*Toxoplasma* IgG commercial assays included in the study.

Assay	Sensitivity mean % (range)	Specificity mean % (range)	PPV mean % (range)	NPV mean % (range)	Comments
Access Toxo IgG II^®^	89.7	100	100	92.8	
	Low IgG: 13.4	Low IgG: 99.6			
Advia Centaur^®^	100	100	100	100	Single study
	Low IgG: 51.1				
Architect Toxo IgG^®^	90.7 (80.8–99.6)	99.8 (99.5–100)	99.7 (99.1–100)	97.3 (94.6–99.5)	For low IgG, one study did not provide crude results [13]
	Low IgG: 30.5 (12–43.9)	Low IgG: 100			
Axsym Toxo IgG^®^	96.1 (90.2–99.6)	99.7 (99.5–100)	99.7 (99.4–100)	97.2 (93.1–99.5)	
BioPlex 2200 ToRCH IgG/IgM^®^	97.8	91.3	98.5	87.5	Single study
Elecsys Toxo IgG^®^	98.6 (97.5–100)	99.6 (98.7–100)	99.2 (98.3–100)	99.4 (98.7–100)	100% concordance with DT for sera with interfering diseases
	Low IgG: 88.6 (73.2–95.6)	Low IgG: 99.6 (99.3–100)			
Immulite 2000 anti-Toxoplasma IgG^®^	93.5 (87.9–99)	99.8 (99.6–100)	99.4	92.7	
Liaison Toxo IgG II^®^	94.8 (93.8–95.8)	99.5 (na)	98.2 (96.8–99.5)	97.4 (95.7–99.1)	One study merged results obtained with low and standard IgG titers (58.9% sensitivity)
	Low IgG: 25.5 (6.7–58.9)	Low IgG: 100			
Platelia Toxo IgG^®^	96.4 (95.6–97.2)	99.4 (98.7–100)	98.4 (96.7–100)	97.7 (97.1–98.3)	In one study, only 29/56 discrepant EIA were confirmed by DT
	Low IgG: 47.6 (32.9–62.2)	Low IgG: 100			
TGS TA Toxo IgG/IDS-iSYS^®^	97	97	nd	nd	
	Low IgG: 46.3	Low IgG: 96.7	Low IgG: 86.4	Low IgG: 79.8	
Vidas Toxo IgG^®^	95.5 (91.4–100)	99.8 (99.5–100)	99.1 (96.8–100)	97.7 (93.9–99.5)	
	Low IgG: 44.2 (12.2–63.3)	Low IgG: 99.7 (99–100)			
Vitros ECiQ Toxoplasma IgG^®^	93.4	100	nd	nd	Single study, no separate analysis of IgG et IgM results
Pastorex Toxo^®^	98.8	98.8	98.8	98.8	Single study, 6.5% false-positive with interfering diseases, sensitivity 97.3% and 100% in acute and chronic toxoplasmosis, respectively
	Low IgG: 51.5				
Toxocell^®^	96.8	97.6	97.6	96.8	Single study, 11% false-positive results with interfering diseases, Sensitivity 97.3% and 100% in acute and chronic toxoplasmosis, respectively
	Low IgG: 66.7				
Toxo HAI^®^	100	99.2	99.2	100	Single study, 4.3% false-positive results with interfering diseases, Sensitivity 97.3% and 100% in acute and chronic toxoplasmosis, respectively
	Low IgG: 97				
Toxolatex^®^	93.7	97.1	97.1	93.7	Single study, 11% false-positive results with interfering diseases, Sensitivity 94.6% and 100% in acute and chronic toxoplasmosis, respectively
	Low IgG: 51.5				
OnSite Toxo IgG/IgM Combo Rapid Test^®^	100	98	98.8	100	Single study
					Detects both specific IgG and IgM
Toxo IgG/IgM Rapid Test ^®^ Biopanda	100	96	98.3	100	Single study
					Detects both specific IgG and IgM
Toxoplasma ICT IgG/IgM^®^ LDBio	100	99.1 (98.6–100)	97.6 (95.8–99.4)	100	Detects both specific IgG and IgM, 100% sensitivity in acute infections, 100% concordance with WB Toxo IgGII
WB Toxo IgGII^®^	Low IgG: 99.2	100	100	99.2	Single study, 99.4% NPV, 100% concordance with DT for equivocal sera and interfering diseases

PPV: positive predictive value; NPV: negative positive value; DT: dye-test; na: not applicable; nd: not determined.

### Specificity

The specificity of IgG assays on routine sera ranged from 91.3% to 100% (Table 2). Thirteen out of 20 assays had specificity >99%, of which 10 were EIA. Specificity for the detection of low IgG titers ranged from 96.7% to 100%, but it was not evaluated in all studies. The apparent lack of sensitivity to detect low IgG titers was balanced by excellent specificity, being equal to or nearly 100% for Architect Toxo IgG^®^, Platelia Toxo IgG^®^, Vidas Toxo IgG^®^, Elecsys Toxo IgG^®^, and Liaison Toxo IgGII^®^. Not surprisingly, methods with the poorest sensitivity in this setting, like the Liaison Toxo IgGII^®^ or the Architect Toxo IgG^®^ assays, had the highest specificity (Table 2).

The possible interference of non-specific immunoglobulins produced in various clinical contexts (auto-immune diseases, viral infections, etc.) was specifically addressed in only two studies [24, 26], which showed that EIA were very specific regarding such serum panels, while agglutination methods were more impacted, leading to 4.3% to 11% false-positive results.

### PPV and NPV

PPV and NPV were reported or could be calculated from 11 studies [5, 7–10, 13, 14, 16, 24, 26] and are presented in Table 2. PPV and NPV varied from 97.1% to 100% and from 87.5% to 100%, respectively, not taking into account studies focusing on low IgG results. Whereas the PPV was very good for most EIA and manual methods, the NPV appeared to be the more discriminant parameter among methods (Fig. 2).

### Kinetics of IgG detection after primary infection

The ability to detect IgG early after primary infection was assessed in few studies [5, 7, 13, 16, 24]. Franck et al. showed on 101 sequential sera obtained during seroconversion, that Elecsys Toxo IgG^®^ did not detect IgG in 8 out of 17 patients who tested positive with WB Toxo IgG II^®^ [5]. Murat et al. compared the results obtained with Vidas, Architect and Liaison on 15 cases of seroconversion, and observed that Vidas and Architect were the last assays to become positive in 5 and 1 cases, respectively [16]. Gay-Andrieu et al. confirmed the longer delay in IgG detection by Vidas, compared to AxSYM and/or Architect in 20 out of 28 cases of seroconversions (74 sequential serum samples) [7]. Interestingly, AxSYM tested positive prior to Architect in 8 out of 28 cases, while Architect tested positive prior to AxSYM in only 2 cases. When considering GZ results as positive, AxSYM tested positive prior to Architect in five cases, and Architect tested positive prior to AxSYM in three cases, while Vidas precocity was hardly improved. In the study by Mahinc et al. comparing the LDBio ICT assay to Architect, the ICT was positive in 16/50 cases, while only IgM were detected with Architect or Toxo-ISAGA (BioMérieux) [13]. Finally, Villard et al. compared the results obtained with several EIAs on a single early serum from 16 seroconversion cases, and showed that Advia Centaur and Elecsys constantly tested negative, while Architect, Platelia and Liaison detected IgG in 2, 2, and 1 cases, respectively [24]. Of note, Architect, Elecsys and Liaison yielded GZ results in 5, 14, and 1 additional cases, respectively.

### Ease of use

The characteristics of the various serologic techniques are listed in Table 3. Overall, EIA assays need electricity and require specific skills and training of users, to ensure high quality results. Particularly, these techniques need calibration and assessment of reagents using negative and positive controls analyzed in each series or at least daily, when analyses are performed on demand on a multiparametric device. Additionally, these automated devices require regular maintenance operations by trained technicians, to ensure reliable results. Similarly, all reagents need cold chain shipment and storage in refrigerators, which can be a limiting factor in some countries. Microplate EIA can be performed manually (serum dilutions and reagents distribution, washing steps) but still need a spectrophotometer, preferably linked to a computer to calculate the results and avoid errors. The use of a microplate setup device is recommended to ensure reproducible results.

**Table 3:** Characteristics of serologic methods used for anti-*Toxoplasma* IgG detection.

Characteristics	Microplate ELISA	Automated Immuno-assay	HAI	AGGLUTINATION	ICT	WB
Multiparametric	No	Yes	No	No	No	No
Turnaround time	1–3 series per week, depending on the lab activity	Series or on demand, depending on the lab activity	Most suited to series	On demand	Unitary test	Unitary test
Suitability for emergency	No	Yes	Possible	Yes	Yes	No
Duration of analysis	3–4 h	0.5–2.5 h	2.5 h	10 min	15 min	3 h
Storage of reagents	4–8 °C	4–8 °C	4–8 °C	4–8 °C	RT	4–8 °C
Device	Spectrophotometer*	Automate	None	None	None	Oscillating agitator
Maintenance	Yearly	Daily, weekly, monthly, yearly	None	None	None	None
Handiness	Skills required^++^	Skills required^++^	Easy	Easy	Easy	Skills required^+^
Reading/interpretation	Standardized	Standardized	Touchy	Easy	Easy	Easy
Cost	Intermediate	High	Low	Low	Low-Intermediate	High
Destination	Reference or district lab	Reference lab	District lab	Local lab	Local lab	Reference or district lab

RT: room temperature.

*These assays can be automated using a microplate setup device.

Agglutination assays are very easy to use and need no apparatus. WB assays are quite simple too, but require an oscillating agitator. All these kits must be kept at +4 to 8 °C.

The storage temperature and staff skills can be limiting factors in favor of immunochromatographic tests, which can be stored at room temperature, and can thus be used in primary care centers of low-income countries.

## Discussion

The choice of a serological method for the diagnosis of toxoplasmosis is important, as it can have clinical consequences. Insight into the advantages and drawbacks of a given assay is crucial to guide complementary tests in some clinical settings, if needed. Other criteria that can help the choice of the assay are robustness, the need to obtain a quick result or to have trained staff, which can differ according to the population screened, the geographic location, and the resources of the laboratory, or the health care policy of the country.

Agglutination tests can be used as complementary techniques in tertiary care laboratories or screening techniques in primary or secondary care centers, since they are easy to use and to implement. However, one must be aware of their limitations. Only four currently marketed assays have been thoroughly evaluated in only one study. It showed that the immuno-hemagglutination assay had the best sensitivity, including for low IgG titers, but this latter point was evaluated only on a small number of sera (*n* = 45) [26]. The latex agglutination assay Toxolatex^®^ had the lowest performances and should be avoided.

In laboratories in high or middle-income countries or possibly in tertiary care hospitals in low-income countries, the EIA assays offer the best performance/cadence ratio, and ensure reagent traceability together with storage of quality control data and maintenance and archiving of patient records on a dedicated server, compatible with the requirements for laboratory accreditation. Usually, standard curves make it possible to define a positive and negative threshold separated by a grey zone of undetermined results. In this context, the WB Toxo IgGII^®^ is a frequently used method to confirm the specificity of GZ results obtained with these EIA techniques, as it showed perfect agreement with the DT gold standard technique, but unlike DT, does not require use of mice as a source of fresh *Toxoplasma* tachyzoites. Although quite easy to perform, this test is expensive and is not suitable for large-scale use as a screening method, but it is now established as a reference technique.

Some EIAs (Bioplex, Advia Centaur and Vitros) benefited from only one evaluation study, and would therefore need to be evaluated further. Particularly, Kasper et al. provided sensitivity and specificity rates on groups of sera known as IgG+/IgM+ or IgG+/IgM−, instead of separate analysis of IgG and IgM using Vitros [11]. This study was not excluded because it was the only one evaluating the Vitros system. The Bioplex assay is based on unique technology, and this first evaluation by Guigue et al. appeared disappointing, but needs confirmation [9]. The TGS/TA IDS-iSYS has a well-defined cut-off value with no GZ, avoiding problems with the interpretation of such results. Importantly, this GZ is the weakness of most EIAs, and manufacturers recommend considering GZ results as negative, since they can correspond to either false-positive or false-negative results. When specific IgM are simultaneously detected, GZ IgG results may correspond to a beginning of seroconversion, which led some authors to propose to lower the positivity threshold for some EIAs [15] or to immediately confirm IgG titers below or within the GZ using the WB Toxo IgGII^®^, to allow earlier diagnosis and treatment of pregnant women. Armengol et al. [1] showed that the WB Toxo IgGII^®^ can detect specific IgG one week before Elecsys Toxo IgG^®^ and 2–3 weeks before most other EIAs. However, without a positive IgM result, this practice is not recommended. Reasonably, we presented only the performances of these assays, considering GZ results as negative, and recalculated authors’ data when necessary, to ensure a fair comparison between all assays. Some authors provided sensitivity, specificity, PPV and NPV values for both situations, i.e. considering GZ results as true-positive or true-negative, and showed huge variations, particularly for sera with low IgG titers [4, 14]. Only two studies addressed specifically the issue of detecting low IgG titers [4, 23]. In this specific situation, apart from the WB Toxo IgG II^®^, the Elecsys Toxo IgG^®^ assay performed fairly well. This ability of Elecsys to detect low IgG amounts may seem contradictory with its delay to detect IgG following primary infection, and is probably linked to the antigenic mix used in this assay, which is probably enriched in parasite cytoplasmic antigens rather than surface antigens.

For all EIAs except Bioplex and TGS/TA, the specificity exceeded 99%, which is not surprising, as these assays usually rely on immunocapture methods. However, Simon et al. recently explored the question of false-positive results obtained with Architect Toxo IgG^®^, and found that 60% were due to cross-reaction with the parasite recombinant proteins GRA7 and GRA8; they suggest that exposure to closely related parasites, such as *Hammondia hammondi* or *Neospora caninum*, could explain these false-positive results [22]. Interfering immunoglobulins are mainly IgM, thus IgG EIAs infrequently suffer from non-specific reactions. In contrast, agglutination methods are more prone to false-positive results due to interfering diseases, such as viral infections and auto-immune diseases, particularly latex agglutination (Pastorex^®^ Toxo, Toxocell^®^ latex, Toxolatex^®^) which detects both IgG and IgM.

Two of the recently developed RDTs showed similar performance characteristics albeit in a single study [8]. The overall sensitivity of the three rapid tests was 100%, but some false-positive results may be observed. They have the advantage of detecting both isotypes allowing diagnosis of recent infection.

The reliability of IgG detection has important clinical implications, as it enables the user to consider a woman as immunized when performed at the beginning of pregnancy, to consider the diagnosis of *Toxoplasma* reactivation in an immunocompromized patient, and to identify seropositive organ donors who could transmit infection to seronegative transplant recipients. In the first situation, the specificity and the PPV are of utmost importance, while in the second situation, the NPV is crucial to exclude the diagnosis. Consequently, PPV and NPV are paramount indicators, depending on the population screened. In low-resource countries facing HIV-associated opportunistic infections, the ideal technique should ally affordability, ease-of-use on bedside, storage at room temperature, and excellent NPV to rule out diagnosis. In this setting, an immunochromatographic test would be best suited, and has the advantage of detecting both specific isotypes, thus affording the diagnosis of recently acquired as well as reactivation infections. For screening of pregnant women at antenatal care centers, both immunochromatographic tests and hemagglutination methods would be suitable to determine the serologic status at the beginning of pregnancy, and provide hygiene recommendations to those who are seronegative.

In high-income countries, the main categories of patients targeted for serological diagnosis are pregnant women and transplant recipients or organ donors. The challenge is to have a technique that provides a positive result with the highest degree of confidence, so that a pregnant woman can be definitively considered immunized and protected. This also allows healthcare professionals to guide chemoprophylaxis according to transplant recipient and organ donor serostatus. In these settings, EIAs which have a high PPV are the most appropriate techniques, all the more so as they are integrated in multiparameter automated systems with high cadence adapted to emergencies. In these patient populations, specific IgM detection is an important complementary parameter to rule out recent infection and guide treatment or patient management (transplantation delay, amniocentesis) [20].

Altogether, the choice of a serological assay to detect anti-*Toxoplasma* IgG must be weighed, and medical biologists should be aware of the pitfalls of their technique and seek advice from a reference laboratory when needed [25]. The pitfalls and advantages of these assays may help to tailor implementation of *Toxoplasma* serologic screening in developing countries as part of essential *in vitro* diagnostics for advanced HIV, as recently advocated by international experts [3].

## Supplementary Material

The supplementary material of this article is available at https://www.parasite-journal.org/10.1051/parasite/2021035. *Supplementary Table 1*: Number of references retrieved according to search terms using PubMed and Web of Science database.

## Conflict of interest

Author declared no conflict of interests.

## Funding

The authors received no specific funding for this work.
