# Supplementary material for: Anti-Toxoplasma IgG assays: What performances for what purpose? A systematic review
Source: Parasite. 2021 Apr 26;28:39. doi: 10.1051/parasite/2021035 (PMC8078101; doi:10.1051/parasite/2021035)
Supplement: Supplementary file 1 — Supplementary Table 1: Number of references retrieved according to search terms using PubMed and Web of Science database. [file parasite-28-39-s1.pdf]

Supplementary Table 1: Number of references retrieved according to search terms using PubMed and Web of Science database.

Database	Search terms	No of articles retrieved	No of articles after refinement	No of articles screened for general objectives
PubMed §	((("Toxoplasmosis/diagnosis"[Mesh]) AND "Serologic Tests"[Mesh])) NOT "Toxoplasmosis, Congenital"[Mesh]	173	na	173
PubMed §	anti-Toxoplasma IgG (title/abstract)	151	na	151
PubMed §	toxoplasmosis serology (title/abstract)	50	na	50
PubMed §	toxoplasmosis assay (title/abstract)	160	na	160
Web of Science#	Toxoplasma serology or anti-Toxoplasma IgG	1798	Refined with “evaluation”<br>Refined with “comparison”<br>Refined with “performance”	153<br>84<br>55
Total				826

§ restricted to 1990-2020 (October 28th), “humans”, “English” and “abstract available”

# restricted to 1990-2020 (October 28th), “humans”, “English” and research areas “parasitology”

na: not applicable
